# Isolation and Characterization of the Diatom *Phaeodactylum* Δ5-Elongase Gene for Transgenic LC-PUFA Production in *Pichia pastoris*

**DOI:** 10.3390/md12031317

**Published:** 2014-03-07

**Authors:** Mulan Jiang, Bing Guo, Xia Wan, Yangmin Gong, Yinbo Zhang, Chuanjiong Hu

**Affiliations:** 1 Key Laboratory of Biology and Genetic Improvement of Oil Crops, Ministry of Agriculture, Oil Crops Research Institute of Chinese Academy of Agricultural Sciences, Wuhan 430062, China; E-Mails: mljiang@oilcrops.cn (M.J.); tcguobing@126.com (B.G.); wanxia@oilcrops.cn (X.W.); gongyangmin@caas.cn (Y.G.); zhangyinbo@caas.cn (Y.Z.); 2 Hubei Key Laboratory of Lipid Chemistry and Nutrition, Wuhan 430062, China

**Keywords:** diatom fatty acids metabolites, *Phaeodactylum* Δ5-elongase, functional stacking of ELO5 and FAD4

## Abstract

The diatom *Phaeodactylum tricornutum* can accumulate eicosapentaenoic acid (EPA) up to 30% of the total fatty acids. This species has been targeted for isolating gene encoding desaturases and elongases for long-chain polyunsaturated fatty acid (LC-PUFA) metabolic engineering. Here we first report the cloning and characterization of Δ5-elongase gene in *P. tricornutum*. A full-length cDNA sequence, designated *PhtELO5*, was shown to contain a 1110 bp open reading frame encoding a 369 amino acid polypeptide. The putative protein contains seven transmembrane regions and two elongase characteristic motifs of FLHXYHH and MYSYY, the latter being typical for microalgal Δ5-elongases. Phylogenetic analysis indicated that PhtELO5 belongs to the ELO5 group, tightly clustered with the counterpart of *Thalassiosira pseudonana*. Heterologous expression of *PhtELO5* in *Pichia pastoris* confirmed that it encodes a specific Δ5-elongase capable of elongating arachidonic acid and eicosapentaenoic acid. Co-expression of PhtELO5 and IsFAD4 (a ∆4-desaturase from *Isochrysis sphaerica*) demonstrated that the high-efficiency biosynthetic pathway of docosahexaenoic acid was assembled in the transgenic yeast. Substrate competition revealed that PhtELO5 exhibited higher activity towards *n*-3 PUFA than *n*-6 PUFA. It is hypothesized that *Phaeodactylum* ELO5 may preferentially participate in biosynthesis of transgenic LC-PUFA via a *n*-3 pathway in the yeast host.

## 1. Introduction

Polyunsaturated fatty acids (PUFAs) are fatty acids of 18 carbons or more in length with two or more double bonds. They can be classified into *n*-6 and *n*-3 major groups, depending on the position of the first double bond proximate to the methyl end of the fatty acid. PUFAs are important structural components that confer membrane fluidity and selective permeability [1,2,3]. Deficiencies in long chain (LC) PUFAs such as DHA and ARA have been associated with disorders in cognitive function, early infancy and various other physiological processes. The *n*-3 PUFAs including DHA and EPA can be used as preventive drugs and supplements [3,4,5,6]. They can be obtained either through diet of marine fish or synthesized from dietary essential fatty acids. However, the marine fish industry is increasingly declining due to overfishing and environmental pollution. It is imperative to develop sustainable and alternative sources of LC-PUFAs [7,8]. One strategy is to make use of high-efficiency desaturase and elongase genes for transgenic production of LC-PUFAs [7].

The fatty acid desaturases and elongases play key roles in the processes of the conventional Δ6-pathway, alternative Δ8-pathway and microbial Δ4-pathaway [8,9]. The ω-3 (or *n*-3) LC-PUFA biosynthesis can start with ALA as substrate which can be produced by some yeasts and plants. The biosynthesis of DHA from ALA involves a series of desaturation and elongation reactions catalyzed by various desaturases and elongases. Δ5-elongase and Δ4-desaturase are rate-limiting enzymes for DHA biosynthesis [2,9]. Molecular characterizations of desaturase and elongase in transgenic organisms were explored with species of yeast, microalgae and plants [9,10,11]. Not a few desaturase genes have been characterized from species of mosses, algae, fungi and others [12,13,14,15,16,17,18]. There are few reports about the Δ5-elongases in *Pavlova salina*, *Marchantia polymorpha* and *Thraustochytrium* sp. [19,20,21,22,23]. Nevertheless, so far the Δ5-elongase of *Phaeodactylum tricornutum* has not been characterized. This diatom is rich in *n*-3 PUFAs with superior genetic resources revealed by genomic research [24,25]. We aimed to characterize the diatom elongase and desaturase which can be used for LC-PUFA metabolic engineering. In this study, we describe the isolation and characterization of Δ5-elongase gene from *Phaeodactylum*, and the functional stacking of Δ5-elongase and Δ4-desaturase in transgenic yeast realizing efficient formation of DHA.

## 2. Results

### 2.1. Isolation of Gene Encoding Δ5-elongase from *P. tricornutum*

NCBI search identified a sequence of *P. tricornutum* mRNA, XM_002176650.1 that predicted a hypothetical protein XP_002176686.1. NCBI conserved domain database (CDD) analysis indicated that this shortened protein belonged to the ELO-super family, sharing 53% identity with the closest homologue in *T. pseudonana*. In order to isolate the full length cDNA and the putative gene, primers (Table 1) were designed for amplification of target gene and coding sequence using the High Fidelity PCR system (Roche). The PCR fragments were fully sequenced and assembled. The contig1 of 1241 bp was identified from cDNA as a coding sequence containing an open reading frame (ORF) of 1110 bp in length; and the contig2 from genomic DNA is 1661 bp long containing a partial 5′-UTR (415 bp) and a putative structural gene (1212 bp) which includes a 102 bp long intron. Sequencing analysis indicated that two sequences of ORF of 1110 bp were identical and designated *PhtELO5*. 

**Table 1:** Microbial strains, plasmids and primers.

Strain, Plasmid or Primer	Characteristic, Use and Source
**Strains**	
*E.coli* Top10	*E.coli* host; for DNA manipulations, Transgene (Beijing, China)
*P. pastoris* GS115	his4; Invitrogen (Invitrogen China Limited, Beijing, China)
PHC01	GS115 transformed by empty pHBM906 vector, as control
PHE5.01	GS115 carrying pHBM-PtELO5, for gene expression
PHE5d.01	GS115 carrying pHBM-PtELO5-Δ1, for gene expression
PHE5d.02	GS115 carrying pHBM-PtELO5-Δ2, for gene expression
PAC01	GS115 carrying pAO815, as control
PAE5.01	GS115 carrying *PtELO5* cassette in pAO815, for expression
PDE01	GS115 carrying *FAD4-ELO5* cascade in pAO815, for co-expression
**Plasmids**	
pMD18-T	T-cloning vector, Ap^r^, Takara; for gene cloning
pHBM906	Ap^r^, transformation vector for *P. pastoris*; stored in our lab
pHBM-ELO5	Ap^r^, PCR fragment containing *PtELO5* coding sequence, generated with primers Ptelo5-U/ Ptelo5-D, cloned into pHBM906
pAO815	Ap^r^, HIS4, *P. pastoris* expression vector with AOX1 promoter and terminator
pAO-FAD4	Ap^r^, PCR fragment containing *IsFAD4* coding sequence, generated with primers ISFAD4E-F/ ISFAD4E-R, cloned into pAO815
pAO-ELO5	Ap^r^, PCR fragment containing *PtELO5* coding sequence, generated with primers PTELO5E-F/PTELO5E-R, cloned into pAO815
pT-ELO5	Ap^r^, PCR fragment containing *PtELO5* coding sequence, generated with primers ELO5BGL-F/ELO5BGL-R, cloned into pMD18-T
pAO-D4E5	Ap^r^, *Bgl*II digested fragment of *PtELO5*ORF subcloned into *BamH*I-digested and dephosphorylated pAO-FAD4
* Primers	
Ptelo5-U1	5′- GGGAGACCAGATGGTCGACG-3′
Ptelo5-U2	5′- TCGCGATACCCCGAATATAT-3′
Ptelo5-U3	5′- CAGTTGTCCCTTCAGAACAGC-3′
Ptelo5-U4	5′- TCGTGTAGAAGAGCGTGGCG-3′
Ptelo5-D1	5′- GCTCTGTAATATAGTGCTCTG-3′
Ptelo5-U	5′- GTCATGTGTGGTCCCACAGATACAG-3′
Ptelo5-D	5′- GGCCACTACGARAGACCGGTCATC-3′
Ptelo5-del1F	5′- GTCgatccacccgtgccctctct-3′
Ptelo5-del2F	5′- GTCTTGCACAACTGGAAGGTTC-3′
ISFAD4E-F	5′- CCGCCGGAATTCGCCATGTGCAACGCGGCAGTCG-3′
ISFAD4E-R	5′- CCGCCGGAATTCTCAATCCGCCTTGAGCGTCTC-3′
PTELO5E-F	5′- CCGCCGGAATTCGCCATGTGTGGTCCCACAGATAC-3′
PTELO5E-R	5′- CCGCCGGAATTCCTACGAAGACCGGTCATCCC-3′
ELO5BGL-F	5′- GGAAGATCTAACATCCAAAGACGAAAGG-3′
ELO5BGL-R	5′- GGAAGATCTGCACAAACGAACGTCTCAC-3′
Co-F	5′- GCTCATGATCAACGGGCTCTACCA-3′
Co-R	5′- TCCCCACACTGCGAAGACACCTAC-3′
5′AOX1	5′- GACTGGTTCCAATTGACAAGC-3′
3′AOX1	5′- GCAAATGGCATTCTGACATCC-3′

* Note: Primers Ptelo5-U1 to U4 and -D1 were used for PCR amplifications against putative PhtELO5 structural gene and cDNA; Ptelo5-U, -del1F, -del2F, and -D for PCR-cloning of the full length or truncated PhtELO5 into pHBM906; ISFAD4E-F and ISFAD4E-R for making vector pAO-IsFAD4; PTELO5E-F and PTELO5E-R for making pAO-PhtELO5; ELO5BGL-F and ELO5BGL-R for making ELO5 expression cassette and the subsequent gene stacking cascade; Co-F,Co-R,5′AOX1 and 3′AOX1 in combination with others for PCR and sequencing confirmation of constructs.

### 2.2. Properties of Putative Elongase PhtELO5

**Figure 1:**
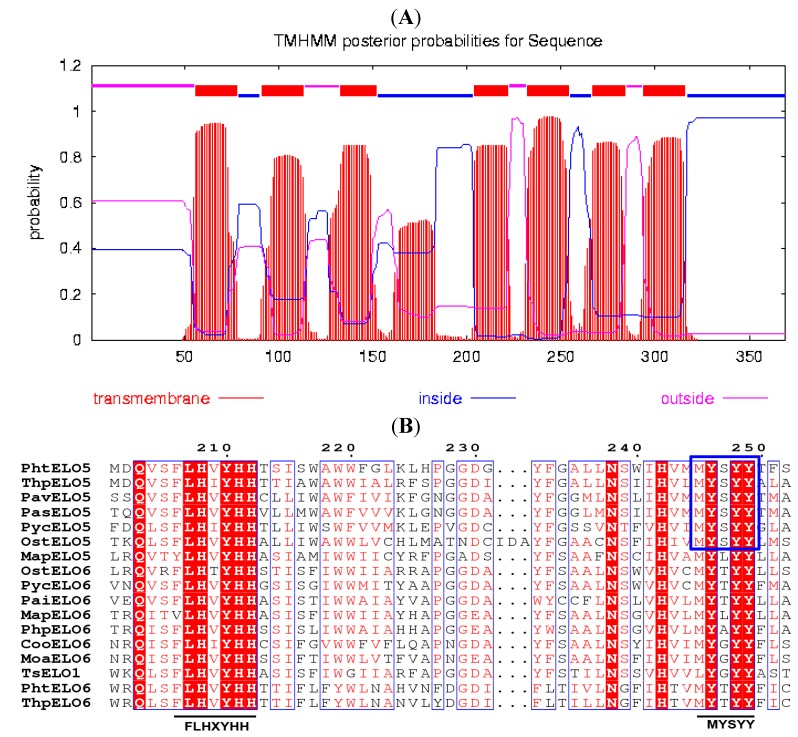
Properties analysis of PhtELO5. (**A**) Prediction of seven transmembrane helices in PhtELO5 by online analysis of TMHMM program (Trans-Membrane prediction using Hidden Markov Models). The predicted regions of transmembrane helices are shown in red, other regions are predicted to be either inside (in blue) or outside (in pink) the membrane. (**B**) Multiple peptide sequence alignment (partly shown) was performed using the Clustal W and ESPript 3, highlighting the typical motifs of Δ5-elongase FLHXYHH and MYSYY which are underlined and boxed. Protein sequences used are the same as that in Figure 2.

**Figure 2:**
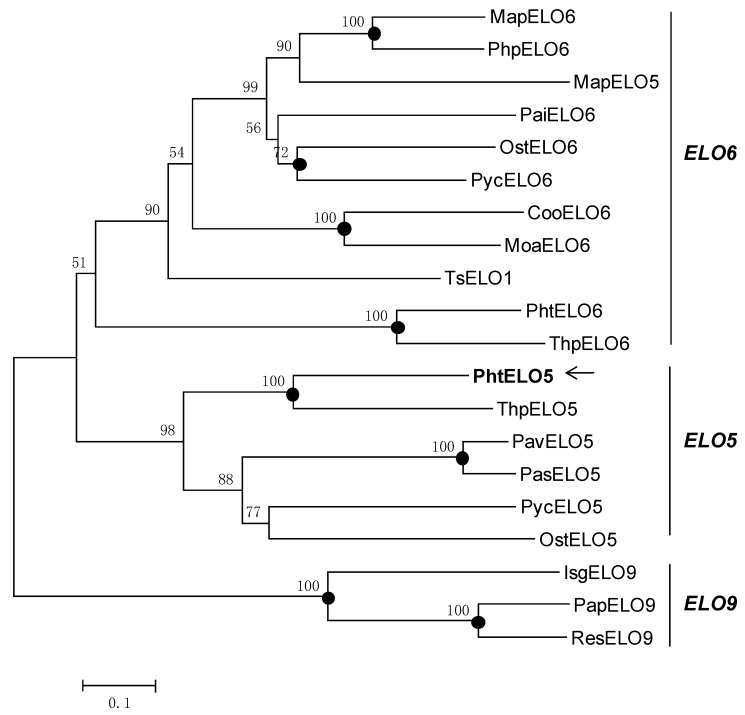
Phylogenetic dendrogram of fatty acid elongase families. The evolutionary relationship was inferred using the Neighbor-Joining method. The percentage of replicate trees in which the associated taxa clustered together in the bootstrap test (1000 replicates) are shown above the branches. Filled circles at nodes indicate phylogenetic branches that were also recovered by using maximum-parsimony algorithms. GenBank accession numbers of the sequences are ABR67690 (PavELO5; *Pavlova viridis*), AAV33630 (PasELO5; *Pavlova* sp. CCMP459), ACR53360 (PycELO5; *Pyramimonas cordata*), BAE71129 (MapELO5; *Marchantia polymorpha*), AAV67798 (OstELO5; *Ostreococcus tauri*), AAV67800 (ThpELO5; *Thalassiosira pseudonana*), AAT85662 (MapELO6; *Marchantia polymorpha*), AAW70157 (PtELO6; *Phaeodactylum tricornutum*), XP_003074750 (OstELO6; *Ostreococcus tauri*), AAV67799 (ThpELO6; *Thalassiosira pseudonana*), ACK99719 (PaiELO6; *Parietochloris incisa*), XP_001780388 (PhpELO6, *Physcomitrella patens*), AEA07666 (CooELO6; *Conidiobolus obscurus*), ACR53359 (PycELO6; *Pyramimonas cordata*), ADE06662 (MoaELO6; *Mortierella alpina*), ADN94475 (PapELO9; *Pavlova pinguis*), ADN94476 (ResELO9; *Rebecca salina*), AAL37626 (IsgELO9; *Isochrysis galbana*). TsELO1 (*Thrasutcohytrium* sp. ATCC26185, ref 22); PtELO5 (*Phaeodactylum tricornutum*, arrow shows this study).

The translated peptide of *PhtELO5* was analyzed by protein family (Pfam) search, Clustal W and TMHMM programs. The results indicated that elongase PhtELO5 is an ELO-family protein containing seven transmembrane regions and two characteristic motifs FLHXYHH and MYSYY. The motif MYSYY is common in all Δ5-elongases analyzed indicating that it may be unique for ELO5 elongases (Figure 1). These properties suggested that putative gene *PhtELO5* is likely to encode fatty acid Δ5-elongase. Phylogenetic analysis indicated that Δ5-, Δ6- and Δ9-elongases included were generally grouped into respective ELO5-, ELO6- and ELO9 clusters in accordance with their functions. However, there are some exceptions, for example, *Marchantia polymorpha* elongase MapELO5 and *Thrasutcohytrium* elongase ELO1 were grouped into ELO6 cluster (Figure 2). Among microalgal Δ5-elongases, PhtELO5 was strongly clustered with *T. pseudonana* Δ5-elongase (ThpELO5) forming a tight clade but quite different from other ELO5s. Protein sequence distances analyzed using DNA-Star (Lasergene) indicated that PhtELO5 shares the highest identity of 51% with ThpELO5 and low identities of 32.6%–38.2% with other members within the ELO5 group. Interestingly, although *Thrasutcohytrium* elongase TsELO1 shares the lowest identity of 26.1% with PhtELO5, it reportedly possessed the activity of ELO5. This may be explained by the existence of motif MYSYY in the TsELO1 protein sequence. Together, it is likely that *PhtELO5* encodes Δ5-elongase and its evolution history is largely different from those of other microalgae and protists [20,22,23]. 

### 2.3. Functional Analysis in Yeast: Confirmation of PhtELO5’s Activity as Δ5-Elongase

**Figure 3:**
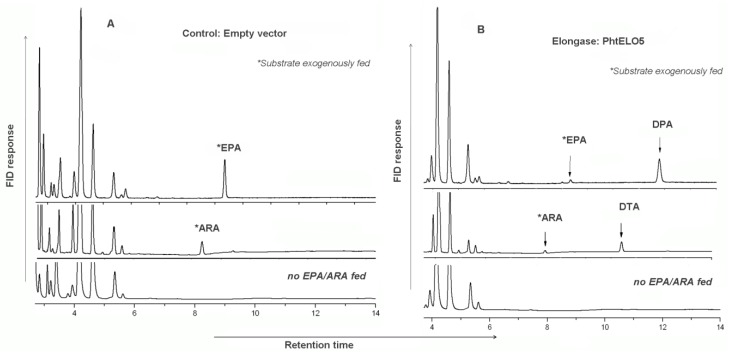
Comparison of fatty acid profiles of the control and PhtELO5-expressing *Pichia* cells. The representative control strain PHC01 (transformed with empty vector pHBM906) and representative ELO5-expressing strain PHE5.01 were grown for 3 days with or without adding substrate and subjected to FA analysis. Samples of 100 µM of (Δ5-) C20 PUFAs were exogenously fed as substrates. (**A**): control strain PHC01; (**B**): PhtELO5-expressing strain PHE5.01. Fatty acid profiles of immediate product from substrate were clearly observed in gene expressed cells but not in controls. Stars indicate substrates which were initially fed and detected as left-over in samples.

**Figure 4:**
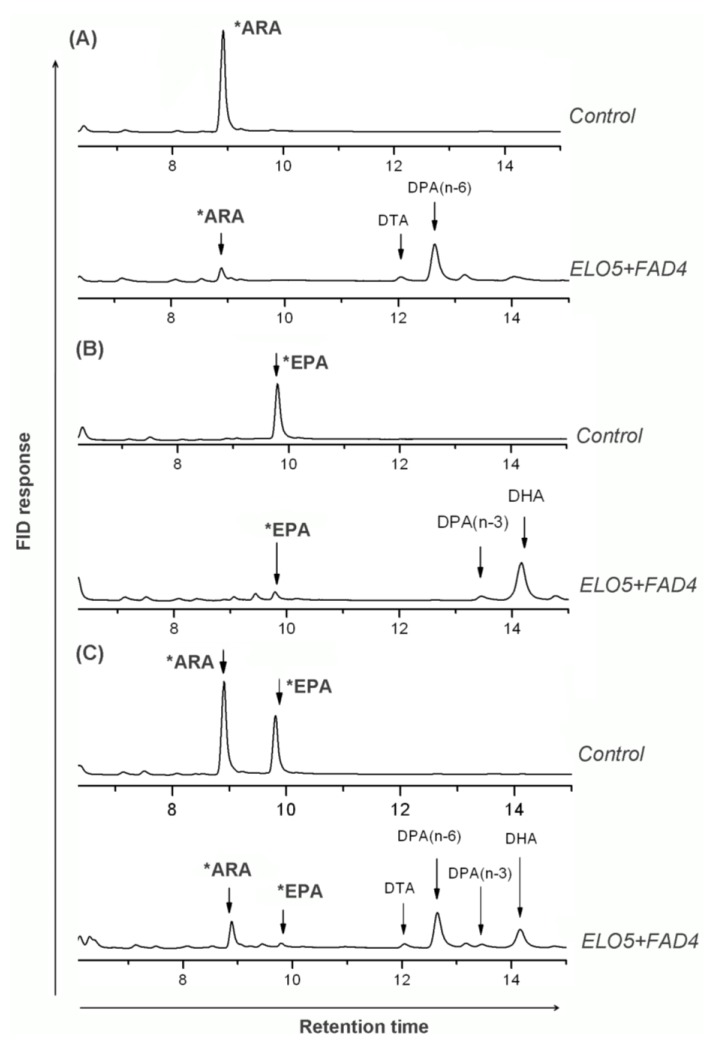
Comparison of fatty acid profiles (partly shown) of the control and co-expressed *Pichia* cells fed with substrates. Control strain PAC01 (transformed with empty vector pAO815) and co-expressed strain PDE01 (transformed with pAO-D4E5) were fed with 100 µM of (Δ5-) C_20_ PUFAs and grown for 3 days, followed by FA analysis. Shown are fatty acid GC profiles of indicated strains fed with ARA (**A**), EPA (**B**) and ARA & EPA (**C**). Fatty acid profiles of immediate product from substrate were clearly observed in co-expressed cells but not in controls. Stars indicate substrates which were initially fed and detected as left-over in samples.

To verify whether the putative PhtELO5 has Δ5-elongase activity or not, we heterologously expressed this gene in *P. pastoris*. ELO5 expression vector was constructed by using plasmid pHBM906 which allows ELO5 ORF to be driven by the inducible AOX1 promoter [26]. Transgenic yeast cells expressing the PhtELO5 ORF were fed with precursor fatty acids (Δ5-) C_20_ PUFAs (referring to ARA and EPA [9,12]) and then were analyzed for fatty acid profiling. Clearly, control strain with empty vector pHBM906 saw no new fatty acids formed, whereas the ELO5-expressing cells fed with ARA or EPA were shown to have a detectable amount of DTA or DPA accordingly (Figure 3 and Figure 4, and Table 2). This result indicated that upon addition of (Δ5-) C_20_ PUFA substrate new fatty acids of DTA and DPA were formed as immediate products. However, it appeared that PhtELO5 displayed a higher rate of converting EPA into DPA(*n*-3) (93.1%) than that of converting ARA into DTA (79.4%). The difference in efficiency was confirmed by using another expression vector pAO815, which exhibited the rate of 90.8% and 83.2%, respectively (Table 3). It is likely that the difference in conversion efficiency may reflect PhtELO5’s substrate preference. 

**Table 2:** Fatty acid composition of transgenic *P. pastoris* GS115.

Fatty Acid Composition (% of Total Fatty Acids)	*P. pastoris* with Plasmids							
	PAC01(E)	PDE01(E)	PAC01(A)	PDE01 (A)	PAC01(EA)	PDE01(EA)	PAC01(−)	PDE01(−)
C14:0	9.75 ± 0.13	15.56 ± 0.23	9.77 ± 0.11	16.2 ± 0.09	10.12 ± 0.24	17.12 ± 0.23	8.98 ± 0.19	13.79 ± 0.24
C16:0	3.01 ± 0.13	10.08 ± 0.32	2.98 ± 0.15	9.90 ± 0.14	3.11 ± 0.18	9.29 ± 0.27	3.48 ± 0.12	9.25 ± 0.21
C17:0	1.01 ± 0.07	1.24 ± 0.04	1.23 ± 0.05	1.13 ± 0.15	1.31 ± 0.13	1.47 ± 0.09	1.36 ± 0.09	1.09 ± 0.04
C17:1	2.90 ± 0.18	1.13 ± 0.15	3.01 ± 0.17	1.13 ± 0.15	3.32 ± 0.14	1.59 ± 0.11	2.50 ± 0.10	0.81 ± 0.08
C18:0	6.89 ± 0.33	6.03 ± 0.39	7.24 ± 0.32	7.44 ± 0.22	7.54 ± 0.32	6.69 ± 0.23	6.80 ± 0.22	6.43 ± 0.10
C18:1 *n*-9	2.87 ± 0.14	2.31 ± 0.18	2.79 ± 0.12	2.00 ± 0.11	3.07 ± 0.16	2.90 ± 0.19	2.24 ± 0.16	1.77 ± 0.07
C18:1 *n*-7	42.86 ± 2.68	34.04 ± 2.01	39.13 ± 2.05	32.95 ± 2.92	38.55 ± 1.63	29.10 ± 1.92	38.14 ± 2.398	33.46 ± 1.73
C18:2 *n*-6	19.09 ± 0.75	21.31 ± 0.67	18.67 ± 0.33	20.28 ± 0.64	17.27 ± 0.47	17.32 ± 0.69	28.63 ± 1.18	27.22 ± 0.73
C18:3 *n*-3	4.62 ± 0.13	4.39 ± 0.19	4.68 ± 0.19	4.07 ± 0.13	4.16 ± 0.23	4.14 ± 0.16	6.14 ± 0.43	4.69 ± 0.08
C20:0	1.79 ± 0.19	1.32 ± 0.23	1.26 ± 0.23	1.67 ± 0.12	1.74 ± 0.14	1.66 ± 0.11	1.72 ± 0.08	1.51 ± 0.05
C20:4 *n*-6 (ARA)	ND	ND	7.82 ± 0.23	0.54 ± 0.04	3.32 ± 0.17	1.56 ± 0.05	ND	ND
C20:5 *n*-3 (EPA)	5.27 ± 0.02	0.26 ± 0.02	ND	ND	5.31 ± 0.02	0.30 ± 0.02	ND	ND
C22:4 *n*-6 (DTA)	ND	ND	ND	0.24 ± 0.01	ND	0.30 ± 0.02	ND	ND
C22:5 *n*-6 (DPA)	ND	ND	ND	2.44 ± 0.03	ND	3.00 ± 0.07	ND	ND
C22:5 *n*-3 (DPA)	ND	0.24 ± 0.03	ND	ND	ND	0.34 ± 0.01	ND	ND
C22:6 *n*-3 (DHA)	ND	2.35 ± 0.05	ND	ND	ND	1.82 ± 0.03	ND	ND

Note: (1) Cells transformed with empty vector pAO815 (representative strain PAC01) or recombinant vector pAO-D4E5 (representative strain PDE01) grown on different substrate containing media were tested. (2) PAC01(E): Strain PAC01 with EPA; PDE01(E): Strain PDE01 with EPA; PAC01(A): Strain PAC01 with ARA; PDE01(A): Strain PDE01 with ARA; PAC01(EA): Strain PAC01with EPA and ARA; PDE01(EA): Strain PDE01 with EPA and ARA; PAC01(−): Strain PAC01 without adding substrate; PDE01(−): Strain PDE01 without adding substrate. ND: not detected or not detectable.

**Table 3:** Substrate conversion rates by FAD4-ELO5 co-expressed transgenic yeast.

	Conversation Rate (%)	
	Addition of Single Substrate	Addition of Double Substrates
EPA→DPA(*n*-3)	90.8	87.9
DPA(*n*-3)→DHA	90.8	84.4
ARA→DTA	83.2	67.9
DTA→DPA(*n*-6)	90.9	90.9

**Figure 5:**
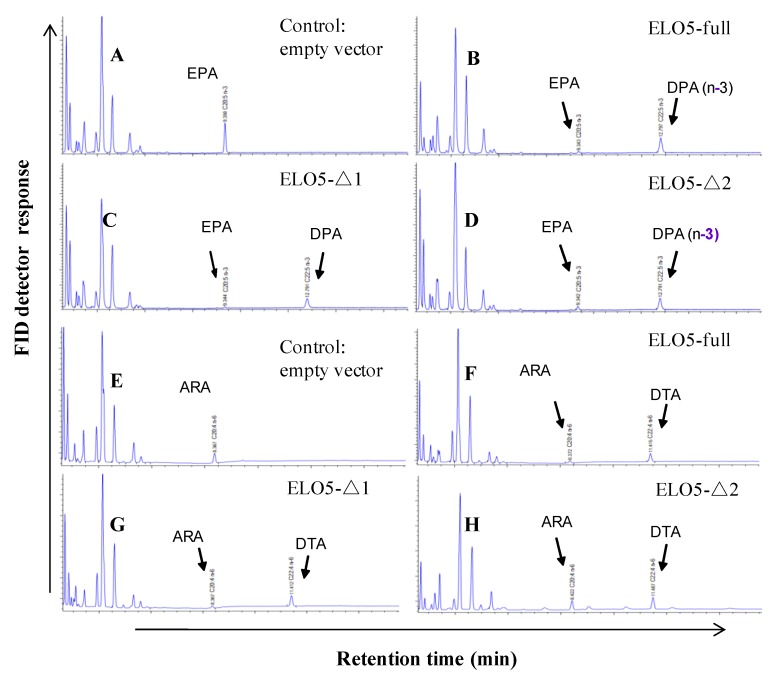
Fatty acid profiling patterns of transgenic *Pichia* cells expressing various versions of PhtELO5. Yeast cells transformed with empty vector pHBM906, and expression vectors containing ELO5-full length, ELO5-Δ1 and ELO5-Δ2 were fed with (Δ5-) C_20_ PUFAs and analyzed for FA composition. (**A**) to (**D**): indicated strains fed with EPA; (**E**) to (**H**): indicated strains fed with ARA.

Based on domain analysis of PhtELO5, we carried out domain deletion experiments to verify the function of key ELO-conserved regions. Yeast cells expressing the truncated ELO5 deleted for 4VR region (4-vinyl reductase, ELO5-Δ1) or ELO5 deleted for 4VR region plus part of the first transmembrane helix (ELO5-Δ2) were shown to have no alteration in fatty acids profiling (Figure 5). The results indicated that at least in transgenic yeast, deletion of *N*-terminal 4VR domain and partial transmembrane regions had little influence on the activity of Δ5-elongase. This is in support of the prediction that PhtELO5’s core activity regions range from amino acid 90–328. Together, it is concluded that PhtELO5 indeed possesses the activity of Δ5-elongase as expected. 

### 2.4. Co-expression of PhtELO5 and IsFAD4 Assembled Function of EPA and DHA Biosynthetic Pathway in Transgenic Yeast

Having confirmed the specificity of PhtELO5 in yeast by single gene expression, we wanted to know further what its performance would be in an assembled EPA and DHA biosynthetic pathway. We thus co-expressed microalgal Δ5-elongase and Δ4-desaturase in yeast to see if they were functional in a non-native system. The co-expression vector was successfully constructed using the stable vector pAO815 [26], which allowed PhtELO5 and IsFAD4 cassettes to be stacked in a cascade through linker sites of *Bgl*II and *BamH*I. The resultant target construct pAO-D4E5 contained ELO5 and FAD4 as a cascade driven by strong inducible promoter pAOX1 (Supplementary Information, Figure S1). 

Yeast can only synthesize the shortest PUFAs like LA and ALA, it needs the addition of (Δ5-) C_20_-PUFAs to continue the DHA biosynthesis via microbial Δ4-pathway [9]. Control strain PAC01 and ELO5-FAD4 co-expressed strain (PDE01) were exogenously fed with ARA and/or EPA. Fatty acid analysis indicated that samples of control cells were unable to detect any new fatty acids formed. In contrast, samples of strain PDE01 expressing ELO5 and FAD4 were new fatty acids detected upon the addition of (Δ5-) C_20_-PUFAs (Figure 4 and Table 2). For instance, fed with ARA alone, strain PDE01 was shown to produce DTA and *n*-6 DPA in comparison with control samples where no new fatty acids identified; this indicated that co-expression indeed led to the conversion of single substrate ARA into intermediate product DTA and the latter subsequently into product *n*-6 DPA (Figure 4A). Fed with single substrate EPA, *n*-3 DPA and DHA were identified as new constituents besides the left-over EPA in FAME samples of strain PDE01. In this case, product *n*-3 DPA was formed from EPA and subsequently transformed into DHA (Figure 4B). Consistently, fed with ARA and EPA as dual substrates, four above mentioned products and two left-over substrates were all detected in PDE01 FAME samples. Taken together, this suggested that the co-expression successfully reconstituted the function of both Δ5 elongation and Δ4 desaturation orientating the *n*-3 pathway. 

### 2.5. PhtELO5’s Converting Efficiencies Varied with Substrates

To test the effect of substrate competition, cells of double gene co-expressed strain PDE01 were fed with the same molar concentration of ARA and EPA concurrently. FA compositions and converting rates were determined to relatively estimate the enzymatic activity or preference upon type of (Δ5-) C_20_-PUFA substrates. As revealed in Table 2 and Table 3, in the presence of single substrate, the rates of conversion EPA→DPA (*n*-3), DPA (*n*-3) →DHA, ARA→DTA and DTA→DPA (*n*-6) were 90.8%, 90.8%, 83.2% and 90.9%, respectively. However in the presence of dual substrates, the rate of conversion (ARA→DTA) dropped drastically from 83.2% to 67.9%, while conversion rates of other substrates showed little change, ranging from 84.4% to 90.9%. The relatively lower conversion rate upon ARA was also observed in ELO5 single gene expression in strain PHD5.01 (79.4% *versus* 93.1%). Obviously, compared to EPA, fewer molecules of ARA were incorporated with elongase PhtELO5. It is interpreted that PhtELO5 is likely to have preference for EPA among (Δ5-) C_20_-PUFA substrates. This finding is consistent with the conclusion that Δ5-elongase is one of the rate-limiting enzymes involved in LC-PUFA biosynthesis [2,25]. 

## 3. Discussion

Diatoms are successful groups of unicellular eukaryotic algae playing important roles in global carbon and silica pools. *P. tricornutum* is one of the most widely utilized model systems for studying the ecology, physiology, and molecular biology of diatoms. Although the genome of this model diatom has been sequenced, the current knowledge on fatty acid metabolic organization and biochemistry remains fragmentary [24,25]. Since the availability of the genome, the PUFA synthesis associated enzymes such as thioesterases, elongases, desaturases, acyl-CoA synthetases and acyltransferases have been increasingly explored [19,23,25]. However, *P. tricornutum*’s fatty acid Δ5-elongase has not been investigated. We therefore conducted the identification and function analysis of *P. tricornutum* Δ5-elongase.

We present herein three lines of evidence verifying that PhtELO5 functions as fatty acid Δ5-elongase. First, sequence analysis indicated that ELO5 is an ELO-family protein, having two typical motifs of FLHXYHH and MYSYY. The motif MYSYY is the typical characteristic for all microalgal Δ5-elongases reported; and it may be crucially required for its enzymatic activity. It is supported by the properties of *Thrasutcohytrium* TsELO1. Although the protein sequence of TsELO1 is largely distant from most Δ5-elongases, it possesses quite high activity of C20-Δ5 elongase [22]. This is likely due to the existence of motif MYSYY. Second, results of PhtELO5 single and double gene expression in yeast not only verified that PhtELO5 indeed possesses the activity of Δ5-elongase, but also demonstrated that gene stacking of microalgal Δ5-elongase and Δ4-desaturase can reconstitute the function of the EPA and DHA biosynthetic pathway in the yeast host. Upon addition of (Δ5-) C_20_-PUFA substrates, both ELO5 single- and double-expression strains were able to form new fatty acids of C_22_ PUFAs, strongly suggesting that PhtELO5 was a key player in orienting DHA synthesis viaΔ4-pathway [9]. Third, PhtELO5 was shown to have higher conversion efficiencies upon EPA than ARA, implying its preference for *n*-3 C_20_-PUFA. This observation is quite different from ELO5 of *Pavlova* and *Thalassiosira* which appeared to have little differences on (Δ5-) C_20_-PUFA substrates [20,21]. To check if PhtELO5 would have activity on saturated C_20_ fatty acid, we did the C20:0 feeding experiment and the results demonstrated that this saturated fatty acid is not the substrate of PhtELO5 (Supplementary Information, Figure S2). 

In this study a high efficiency of converting (Δ5-) C_20_-PUFA substrates was repeatedly observed in ELO5 single and ELO-FAD4 double expressing cells. This is largely different from ELO5s of other microalgae where the conversion rates were generally less than 50% [12,20]. The high conversion rate may reflect the high enzymatic activity of PhtELO5 and IsFAD4. Initially we wanted to co-express *Phaeodactylum* Δ4-desaturase and Δ5-elongase but failed to get Δ4-desaturase functional; thus we then made use of previously described *I. sphaerica* Δ4-desaturase which had been proven to be efficient in converting substrate DPA [18]. Furthermore, *PhtELO5* and *IsFAD4* were constructed under a strong inducible promoter which may have facilitated the functional stacking of both enzymes in *Pichia*. Gene stacking through a combined construct was proven to have advantages such as fewer selective markers and higher expression level [27,28]. However, such very high conversion efficiencies were probably attributed to the continuous strong inducing condition we applied: cells were batch-fed with methanol every 24 h. Expression patterns of target genes may support the explanation. By using Quantitative real-time PCR, mRNA levels of ELO5 and FAD4 were shown to be steadily high over the period of cultivation, indicating that the target genes were stably expressed at a high level under current inductions (Supplementary Information, Figure S3). All these factors have contributed to maintaining the high activities of Δ5-elongase and Δ4-desaturase in the *Pichia* host. 

**Figure 6:**
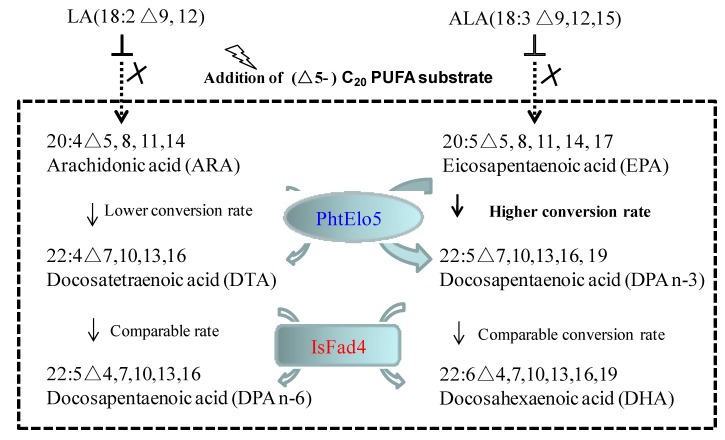
Schematic illustration of the heterologous assembly of microalgal ELO5 and FAD4 implementing EPA and DHA syntheses in transgenic yeast.

In nature, yeast including *P. postoris* cannot synthesize PUFAs longer than LA and ALA [2,9]. Introduction of microalgal ELO5 and FAD4 into yeast cells can reconstruct the functional routes orienting EPA and DHA biosynthesis, as part of the microbial Δ4-pathway (Figure 6). Based on our finding, it is concluded that synthesis of C_22_ PUFAs can be realized via functional assembly of ELO5/FAD4 in the presence of (Δ5-) C_20_ PUFA substrates. ELO5 is likely to have a higher converting activity upon *n*-3 C_20_ PUFA substrate, whereas FAD4 activity remains comparable in both *n*-3 and *n*-6 pathways (Table 3, Figure 6). It is postulated that in transgenic yeast, *Phaeodactylum* ELO5 may determine the *n*-3 pathway as a dominant synthetic route for LC-PUFA production. 

## 4. Experimental Section

### 4.1. Strains and Culture Conditions

The diatom *P. tricornutum* Pt9 (CCMP633) was obtained from the Institute of Hydrobiology, Chinese Academy of Sciences (Wuhan, China). *P. tricornutum* was grown in f/2-enriched artificial sea water (f/2AW, pH8.5) medium at 25 °C with continuous aeration and illumination (50 mol photons m^−2^s^−1^, by cool daylight fluorescent tubes). *P. pastoris* strain GS115 was grown and maintained in YPD medium. 

### 4.2. Sequence Analysis

DNA or protein sequence alignment and neighbor-joining tree construction were done with Clustal W and MEGA 5.2.2. Sequence distances were analyzed by DNA-star. Structural domain prediction was carried out using the online server program TMHMM and ESPript 3. 

### 4.3. Identification and Isolation of the Δ5-Elongase cDNA from *P. tricornutum*

DNA, RNA and cDNA preparation were performed by standard protocols. Molecular tool enzymes and reagent kits were used according to instructions provided by the manufacturers. At the time of study there was only a partial sequence of *P. tricornutumt* mRNA available (NCBI Reference Sequence: XM_002176650.1). This partial start and partial stop fragment was linked to a sequence in chromosome 1. In order to isolate its full length cDNA and putative gene, three forward primers Phtelo5-U1, -U2 and -U3 and one reverse primer Phtelo5-D1 (Table 1) were designed for DNA amplification against genomic DNA and the first strand cDNA. For this purpose, the High Fidelity PCR system (Roche) was employed to minimize the error rate. The specific PCR products were gel purified and cloned into pMD18-T vector (TaKaRa, Dalian, China) after the fragments were prepared by the A-tailing procedure. Inserts were then fully sequenced with complete coverage. Assembly of the sequences allowed the identification of a full length of ORF from cDNA and the coding sequences. It was designated *PhtELO5* and subjected to cloning into yeast expression vectors.

### 4.4. Plasmid Construction and Transformation of Yeast Cells

Plasmids and primers used in this study are featured in Table 1. PCR fragments were cloned into pMD18-T (Takara) and confirmed by sequencing. To characterize the function of ELO5, the *PhtELO5* was cloned into pHBM906 and expressed in *P. pastoris*. Briefly, *PhtELO5* was cloned into pMD18-T through cloning sites of *Not*I and *Cpo*I to generate pMD-ELO5. The full length of *PhtELO5* was released from pMD-ELO5 by *Not*I and *Cpo*I digestions, and subcloned into *Not*I and *Cpo*I-cut pHBM906 (*P. pastoris* expression vector). In the resultant vector pHBM-ELO5, *ELO5* was placed in between the promoter and transcription terminator regions of the AOX1 gene. Linearized by *Sal*I, pHBM-ELO5 was introduced into *P. pastoris* strain GS115 by electroporation (Bio-Rad). Concurrently, the empty pHBM906 vector was introduced into GS115 as control. For co-expression of ELO5 and FAD4 in *P. pastoris*, the expressing vector pAO815 was used to stack the two genes in a cascade. First, IsFAD4 and PtELO5 were individually cloned into pAO815 via *EcoR*I cloning site to form vector pAO-FAD4 and pAO-ELO5, respectively. Then, pAO-FAD4 was digested with *BamH*I followed by dephosphorylation with calf intestine alkaline phosphatase according to the manufacturer’s instructions; and PhtELO5 expression cassette (5′AOX1-PhtELO5-TT) was amplified from vector pAO-ELO5 with primer ELO5BGL-F and ELO5BGL-R using a high fidelity PCR. Finally, after digestion with *Bgl*II, the PhtELO5 cassette was ligated to *BamH*I-digested and dephosphorylated vector pAO-FAD4, resulting in the target vector designated pAO-D4E5. Various PCR and sequencing were carried out to confirm the accuracy of sequence and structure of the stacked cassettes prior to the transformation. 

### 4.5. Heterologous Expression of PtELO5 and IsFAD4 in *P. pastoris*

To verify the function of *PhtELO5*, the recombinant vectors pHBM-ELO5 and/or pAO-D4E5 were introduced in *Pichia pastoris.* The representative strains of three independent positive transformants were selected and grown on 50 mL MGY liquid medium (1.34% YNB with amino acids and ammonium sulfate, 10^−5^% biotin and 1% glycerol) on a shaker (220 rpm) at 28 °C. Cultures of the stationary phase were harvested and washed with sterile deionized water twice and inoculated (at final rate of OD600 = 1) into the 500 mL glass Erlenmeyer flasks containing 50 mL MM medium (1.34% YNB with amino acids and ammonium sulfate, 10^−5^% biotin and 0.5% methanol). Methanol, 0.5% (v/v), was supplemented every 24 h to keep inducing gene expression. All yeast cultures were grown for 72 or 96 h at 20 °C and used for fatty acid analysis. 

Heterologous gene expressions were estimated by Quantitative real-time PCR. Total RNA was isolated from cells using the TRIzol reagent (Invitrogen China Limited, Beijing, China). First-strand cDNA was synthesized using a PrimeScript RT reagent kit with gDNA eraser (TaKaRa Biotechnology (Dalian), Dalian, China). qRT-PCR was performed using iCycler iQ5 real-time PCR system (Bio-Rad, Hercules, CA, USA) and SYBR Premix Ex Taq II (Tli RNaseH plus kit) (TaKaRa Biotechnology (Dalian), Dalian, China), according to the manufacturer’s protocols. Full-length *PhtELO5 or IgFAD4* cDNA was amplified with primer pairs listed in Table 1. The yeast actin gene *ACT1* was used as an internal standard. The relative mRNA level of the target gene was normalized against that of standard. 

### 4.6. PUFA Substrate Feeding

*P. pastoris* transgenic strains carrying various expressing vectors were grown in BMGY medium individually. Each overnight culture was diluted to an OD600 of 0.5 and refreshed by growing for further hours till the OD600 value reached approximately 1.0. The cultures were harvested by centrifugation and resuspended in the same volume of fresh BMGY medium containing 1% NP40, 0.5% methanol and exogenously fed with 100 µM each of (Δ5-) C_20_ FUFAs. The cultures were grown for 72–96 h with addition of 0.5% methanol every 24 h. Cell were harvested, washed once with three volumes of 0.5% Triton X-100, and once with three volumes of distilled water. The pellets were subjected to analysis of fatty acid composition (% of total fatty acids).

### 4.7. Fatty Acid Analysis

Microalgae or yeast cells were dried by the Vacuum Freeze-drying System. Fatty acid methyl esters (FAMEs) were extracted with petroleum ether and trans-methylated with 0.4 M NaOH in methanol [29]. All samples were analyzed using a 7890A gas chromatography (Agilent technologies, Santa Clara, CA, USA) equipped with a flame ionization detector (FID) and an HP-FFAP capillary column (30m × 250 μm × 0.25 μm). High purity nitrogen was used as carrier gas. Standards of fatty acid mixtures were purchased from Sigma-Aldrich (St. Louis, MO, USA). FAMEs were identified by comparison of retention times with those of the authentic standards. The relative amount of FAME was quantified by comparing each peak area with that of the internal standard. 1 μg/μL of behenic acid (22:0) was added into samples as internal standard. The conversion efficiency was calculated as percentage of the relative amount of product divided by the sum of product and substrate (which is left-over), namely conversion rate (%) = [product]/([substrate] + [product]) × 100% [18,30].

## 5. Conclusions

We herein report the characterization and functional analysis of *P. tricornutum* Δ5-elongase gene *PhtELO5*. Heterologous expression in *Pichia* confirmed that ELO5 possesses strong activity of Δ5-elongase capable of elongating (Δ5-) C_20_ PUFA substrates. Substrate competition revealed that PhtELO5 had a higher converting activity towards 20:5*n*-3 than 20:4*n*-6 fatty acids. Functional stacking of *Phaeodactylum* ELO5 and *Isochrysis* FAD4 in *Pichia* reconstituted a high-efficiency biosynthetic pathway leading to transgenic production of DHA. 

## Abbreviations

ARA: arachidonic acid
ALA: α-linolenic acid
DHA: docosahexaenoic acid
DTA: Docosatetraenoic acid
DPA: docosapentaenoic acid
EPA: eicosapentaenoic acid
FA: fatty acid
FAMEs: Fatty acid methyl esters
FID: flame ionization detector
GC-MS: Gas Chromatography-Mass Spectrometry
LA: linoleic Acid
LC: long chain
ORF: open reading frame
PUFA: polyunsaturated fatty acid

## Supplementary Files

Supplementary File 1 Supplementary Information (PDF, 213 KB)
