# Supplementary material for: Isolation and Characterization of the Diatom Phaeodactylum Δ5-Elongase Gene for Transgenic LC-PUFA Production in Pichia pastoris
Source: Mar Drugs. 2014 Mar 7;12(3):1317–34. doi: 10.3390/md12031317 (PMC3967212; doi:10.3390/md12031317)
Supplement: Supplementary File 1 — Supplementary Information (PDF, 213 KB) [file marinedrugs-12-01317-s001.pdf]

Supplementary Information

Figure S1. Schematic illustration of the making of ELO5 and FAD4 stacked construct. IsFAD4 and PtELO5 were individually cloned into pAO815 via *EcoR*I site to form vector pAO-FAD4 and pAO-ELO5, respectively. Vector pAO-FAD4 was digested with *BamH*I followed by dephosphorylation; and PtELO5 expression cassette (5'AOX1-PtELO5-TT) was amplified from vector pAO-ELO5 with primer ELO5BGL-F and ELO5BGL-R using a high fidelity PCR system. The ELO5 cassette with *Bgl*II sites was ligated to *BamH*I-digested and dephosphorylated vector pAO-FAD4 to form the target vector pAO-D4E5, where FAD4- and ELO5-cassettes were placed in same orientation as a cascade.

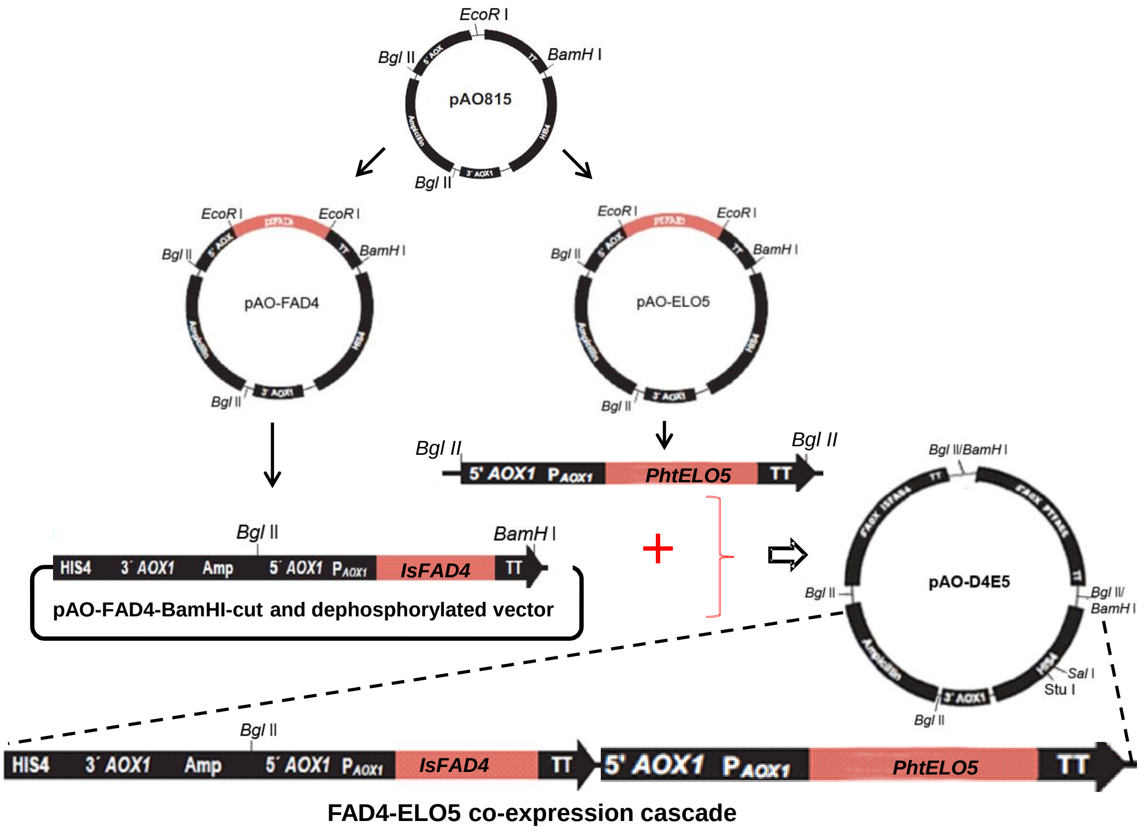

Figure S2. Comparison of fatty acid profiles of the control and PhtELO5-expressed *Pichia* cells fed with saturated C20 FA. ELO5-expressing strain (PHE5.01, upper) and control strain (PHC01, bottom) were grown for 3 days in the presence of 100 µM of C20:0 FA. Shown are fatty acid GC profiles of indicated strains.

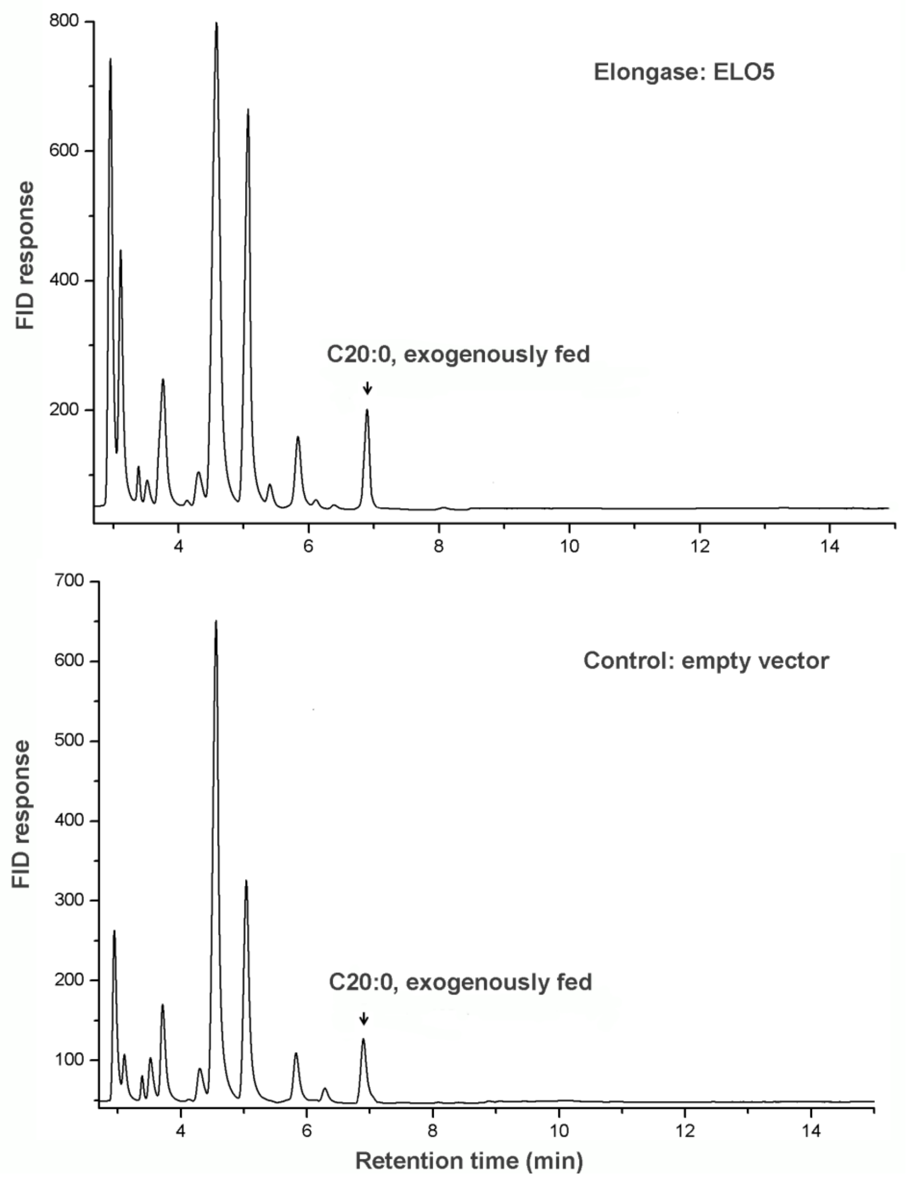

Figure S3. Expression of IgFAD4 and PhtELO5 in co-expressed *P. pastoris*. Strains PAE01 were grown on induction medium containing (Δ5-) C_{20}-PUFA substrates and methanol for 3 days. The daily feeding of 0.5% methanol served to maintain the continuous highly inducing conditions. The cultures were sampled at the stated time points and subjected to RNA preparation and quantitative RT-PCR. Relative mRNA levels of IgFAD4 (**A**) and PhtELO5 (**B**) were normalized to ACT1 transcript level.

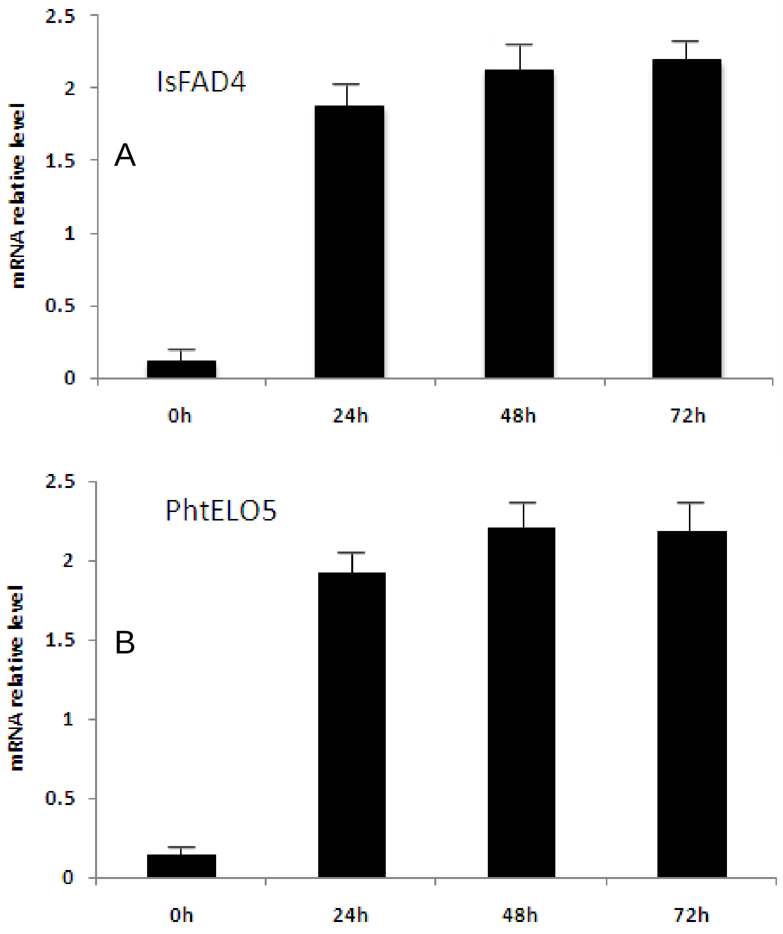

© 2014 by the authors; licensee MDPI, Basel, Switzerland. This article is an open access article distributed under the terms and conditions of the Creative Commons Attribution license (http://creativecommons.org/licenses/by/3.0/).
